# Supplementary figures and images for: The HPV8 E6 protein targets the Hippo and Wnt signaling pathways as part of its arsenal to restrain keratinocyte differentiation
Source: mBio. 2023 Sep 7;14(5):e01556-23. doi: 10.1128/mbio.01556-23 (PMC10653872; doi:10.1128/mbio.01556-23)

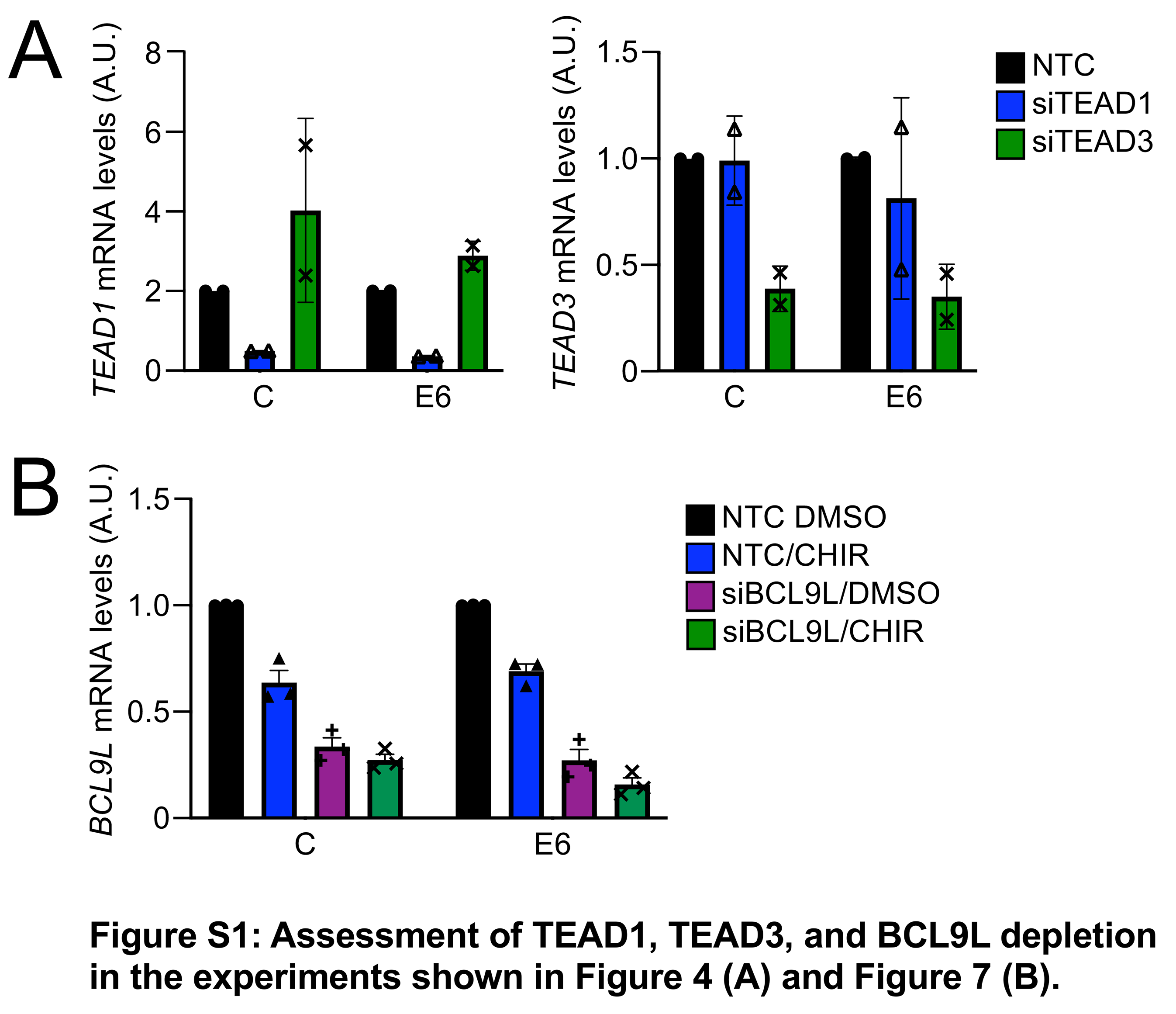

Supplement: Fig. S1 — Assessment of TEAD1, TEAD3, and BCL9L depletion. [file mbio.01556-23-s0001.tif]

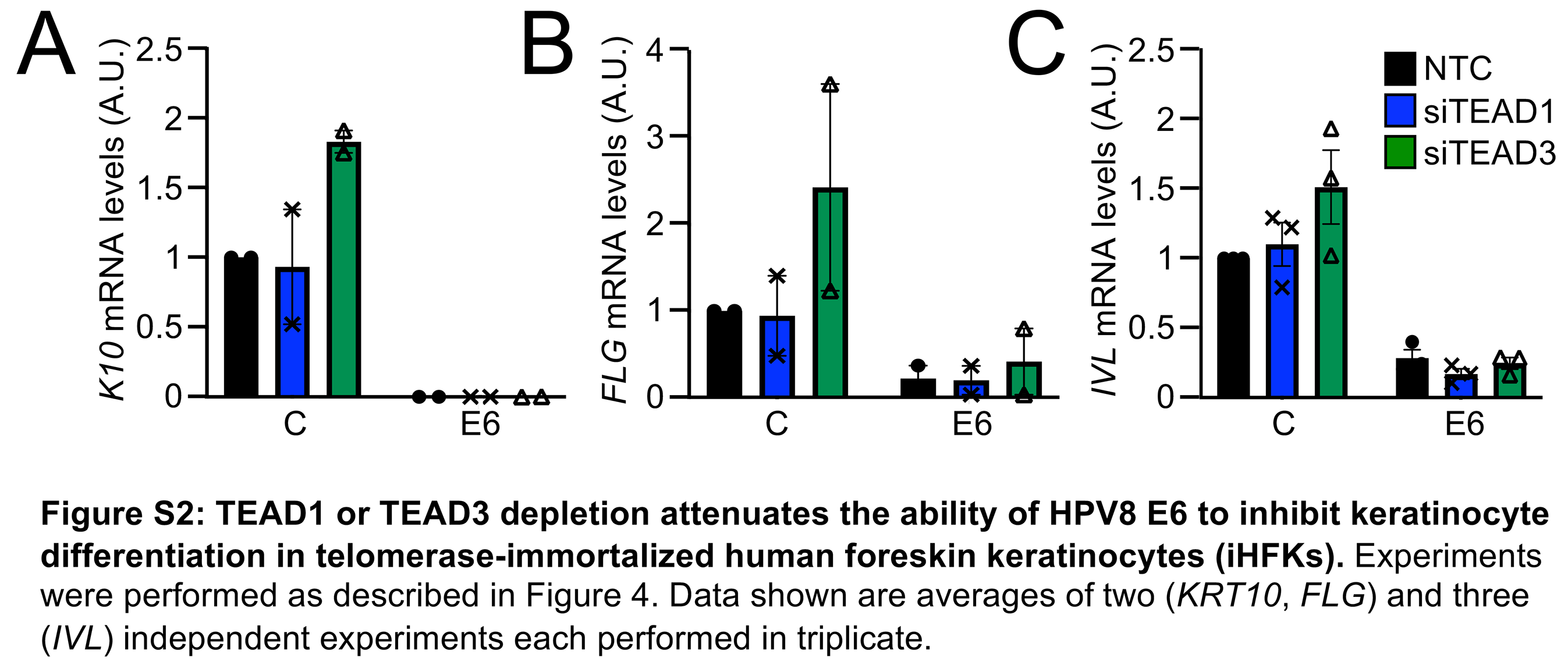

Supplement: Fig. S2 — TEAD1 or TEAD3 depletion attenuates the ability of HPV8 E6 to inhibit keratinocyte differentiation in telomerase-immortalized human foreskin keratinocytes (iHFKs). [file mbio.01556-23-s0002.tif]

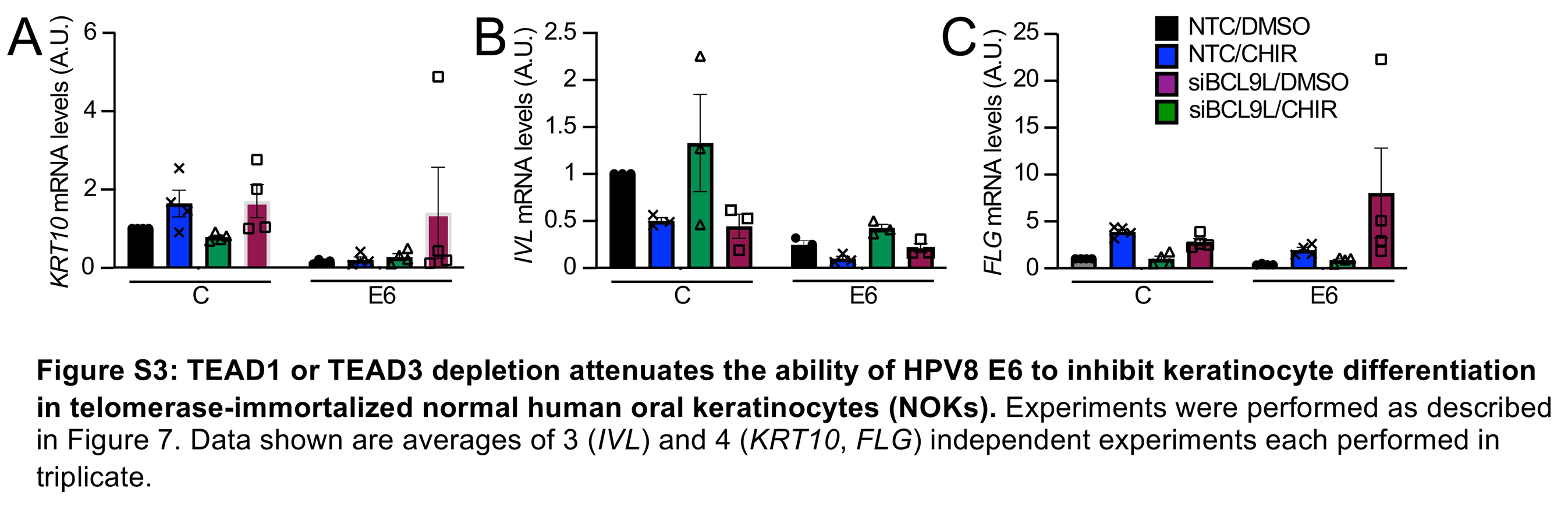

Supplement: Fig. S3 — TEAD1 or TEAD3 depletion attenuates the ability of HPV8 E6 to inhibit keratinocyte differentiation in telomerase-immortalized normal human oral keratinocytes. [file mbio.01556-23-s0003.tif]
