# Supplementary material for: The HPV8 E6 protein targets the Hippo and Wnt signaling pathways as part of its arsenal to restrain keratinocyte differentiation
Source: mBio. 2023 Sep 7;14(5):e01556-23. doi: 10.1128/mbio.01556-23 (PMC10653872; doi:10.1128/mbio.01556-23)
Supplement: Table S1 — HPV8 E6 associated cellular proteins. [file mbio.01556-23-s0004.docx]

**Table S1: Interactors identified by AP/MS**

List of HPV8 E6 interacting cellular proteins identified via affinity purification/mass spectrometry in HCT116 colon cancer cells transfected with HPV8 E6 tagged with FLAG and HA at the C terminus (“HPV8 CE6”) or N-terminus (“HPV8 NE6”). The number of unique peptides (“Unique”) and total peptides (“Total”) are shown.

|  | **HPV8 CE6** | | **HPV8 NE6** | |
| --- | --- | --- | --- | --- |
|  | Unique | Total | Unique | Total |
| AASDHPPT | 6 | 9 | 4 | 6 |
| ADAM15 | 4 | 4 |  |  |
| AGPAT9 | 3 | 3 |  |  |
| AIP | 4 | 4 |  |  |
| AJUBA | 5 | 5 | 4 | 5 |
| AKTIP |  |  | 4 | 6 |
| ARCN1 | 3 | 3 |  |  |
| ARHGAP29 |  |  | 5 | 7 |
| ARVCF | 3 | 3 |  |  |
| AZI1 | 4 | 6 |  |  |
| BCKDHA | 3 | 4 |  |  |
| BCL9 | 4 | 4 |  |  |
| BCL9L | 25 | 31 | 12 | 15 |
| BIRC6 | 7 | 8 | 4 | 5 |
| BOP1 |  |  | 3 | 3 |
| BRF1 | 3 | 4 |  |  |
| BTAF1 | 12 | 13 | 13 | 13 |
| C5orf34 | 8 | 9 | 6 | 6 |
| CALR | 3 | 4 |  |  |
| CAMK2A | 3 | 4 |  |  |
| CAMK2D | 8 | 8 |  |  |
| CBL | 7 | 8 |  |  |
| CCDC101 | 4 | 5 |  |  |
| CCDC6 | 3 | 3 |  |  |
| CD2BP2 | 6 | 8 | 6 | 6 |
| CDC25A | 3 | 3 |  |  |
| CFAP410 | 3 | 3 |  |  |
| CHD3 | 3 | 3 |  |  |
| CHD4 | 18 | 22 | 8 | 9 |
| CIAO1 |  |  | 4 | 8 |
| CREBBP | 106 | 222 | 69 | 147 |
| CSTB | 3 | 3 |  |  |
| CTTNBP2NL | 32 | 55 | 28 | 46 |
| DAP3 |  |  | 7 | 7 |
| DCP1A | 5 | 5 | 3 | 3 |
| DCXR | 5 | 5 | 4 | 4 |
| DDA1 | 3 | 4 |  |  |
| DHCR24 |  |  | 4 | 5 |
| DPYSL2 | 3 | 3 |  |  |
| EBNA1BP2 |  |  | 4 | 5 |
| EHD1 | 4 | 5 |  |  |
| EHD2 | 3 | 3 |  |  |
| ELP4 | 3 | 3 |  |  |
| EMD |  |  | 3 | 3 |
| EPS8L1 | 5 | 5 |  |  |
| ERCC2 | 3 | 3 |  |  |
| EXOSC10 |  |  | 3 | 3 |
| FAM115A | 7 | 9 | 6 | 6 |
| FAM160A2 |  |  | 5 | 7 |
| FAM172A | 4 | 5 |  |  |
| FAM40A | 5 | 5 |  |  |
| FAM83H | 3 | 3 |  |  |
| FAM96B |  |  | 3 | 5 |
| FANCG | 4 | 5 |  |  |
| FOXP4 | 3 | 4 |  |  |
| GAK | 4 | 4 |  |  |
| GALK1 | 3 | 3 |  |  |
| GAPVD1 | 6 | 8 | 3 | 4 |
| GATAD2B | 7 | 7 |  |  |
| GET4 | 3 | 3 |  |  |
| GNA12 | 3 | 3 |  |  |
| GNPAT |  |  | 3 | 4 |
| GPATCH1 | 3 | 3 |  |  |
| GPD2 |  |  | 6 | 7 |
| HDAC2 | 5 | 6 | 3 | 3 |
| HDGFRP2 | 5 | 5 | 3 | 4 |
| HELLS | 5 | 5 | 5 | 5 |
| HJURP | 3 | 3 |  |  |
| HM13 | 3 | 3 |  |  |
| HOOK1 |  |  | 7 | 9 |
| IFT74 | 18 | 20 | 9 | 10 |
| IFT81 | 19 | 19 | 9 | 14 |
| IGBP1 | 3 | 3 |  |  |
| IK | 7 | 7 | 4 | 5 |
| INTS6 | 11 | 15 | 11 | 11 |
| IRAK1 | 3 | 3 |  |  |
| ITPK1 |  |  | 3 | 3 |
| IWS1 | 40 | 81 | 27 | 59 |
| KDM1A | 8 | 9 | 9 | 9 |
| KDM3A | 3 | 3 |  |  |
| KDM3B | 12 | 13 | 6 | 6 |
| KLC1 |  |  | 5 | 5 |
| L2HGDH | 3 | 3 | 3 | 3 |
| LEMD3 | 6 | 7 | 5 | 5 |
| LPP | 4 | 5 |  |  |
| LUC7L2 | 5 | 6 | 5 | 6 |
| LUC7L3 |  |  | 3 | 4 |
| LYAR |  |  | 6 | 6 |
| LZTS2 |  |  | 3 | 4 |
| MAML1 | 34 | 50 | 21 | 27 |
| MAP3K11 | 11 | 11 | 8 | 8 |
| MAP4K2 | 3 | 3 |  |  |
| MBD2 | 8 | 9 | 4 | 4 |
| MED13L |  |  | 4 | 4 |
| MGME1 | 4 | 4 |  |  |
| MOB4 | 3 | 6 |  |  |
| MOCS2 | 3 | 3 |  |  |
| MTA1 | 5 | 6 |  |  |
| MTA2 | 11 | 12 | 4 | 4 |
| MTDH |  |  | 5 | 7 |
| NAB1 | 3 | 3 | 4 | 4 |
| NASP | 4 | 4 |  |  |
| NCAPD3 |  |  | 3 | 3 |
| NCAPG2 | 8 | 8 | 5 | 6 |
| NCBP3 |  |  | 3 | 3 |
| NCOA2 | 3 | 3 |  |  |
| NCOA3 | 19 | 22 | 7 | 7 |
| NCOR2 | 15 | 16 | 3 | 3 |
| NIF3L1 | 13 | 20 | 10 | 17 |
| NLRP2 | 3 | 3 |  |  |
| NLRX1 | 12 | 14 |  |  |
| NOP2 |  |  | 4 | 5 |
| NRIP1 | 10 | 10 | 11 | 11 |
| NUDC | 4 | 4 | 3 | 4 |
| NXN | 5 | 7 |  |  |
| OCRL | 14 | 14 | 15 | 15 |
| OGFR | 4 | 5 |  |  |
| OTUB1 | 6 | 9 | 3 | 3 |
| PARD3 | 5 | 5 | 4 | 4 |
| PDHA1 | 5 | 5 |  |  |
| PDS5A | 3 | 3 |  |  |
| PDZD11 | 4 | 6 | 3 | 6 |
| PEF1 | 6 | 13 | 8 | 17 |
| PER1 |  |  | 3 | 4 |
| PFDN1 | 7 | 9 | 4 | 6 |
| PFDN2 | 9 | 15 | 8 | 15 |
| PFDN4 | 5 | 8 | 4 | 6 |
| PFDN5 | 7 | 11 | 4 | 6 |
| PFDN6 | 9 | 12 | 5 | 6 |
| PIPSL | 3 | 4 |  |  |
| PLCD3 | 5 | 6 | 6 | 7 |
| POLG | 5 | 5 |  |  |
| POLK | 4 | 5 |  |  |
| POP1 |  |  | 5 | 6 |
| PPIH |  |  | 3 | 3 |
| PSD3 | 4 | 4 |  |  |
| PTCD3 | 6 | 6 | 10 | 11 |
| PXN | 3 | 4 |  |  |
| PYGO2 | 6 | 8 |  |  |
| RAI1 | 9 | 18 | 8 | 11 |
| RCOR1 | 6 | 6 | 3 | 5 |
| RECQL4 | 5 | 5 | 5 | 6 |
| RESF1 | 5 | 6 | 6 | 7 |
| RIC8A | 4 | 6 |  |  |
| RIN1 | 4 | 4 | 5 | 5 |
| RIPK2 | 3 | 3 |  |  |
| RNF41 | 3 | 3 |  |  |
| RPS6KA5 | 7 | 7 | 4 | 5 |
| RRBP1 |  |  | 3 | 5 |
| RTF1 | 15 | 20 | 8 | 8 |
| SART1 |  |  | 5 | 5 |
| SATB2 | 3 | 3 | 5 | 5 |
| SCAF8 | 3 | 3 |  |  |
| SDE2 | 8 | 8 | 5 | 6 |
| SHC1 | 3 | 3 |  |  |
| SHROOM2 | 4 | 4 |  |  |
| SIK3 | 18 | 18 | 15 | 20 |
| SKA3 | 10 | 12 | 8 | 10 |
| SMAD2 | 5 | 6 | 4 | 5 |
| SMAD3 | 7 | 10 | 7 | 12 |
| SMAP | 3 | 3 |  |  |
| SMARCAD1 | 3 | 3 | 5 | 5 |
| SMARCD2 | 5 | 5 | 3 | 3 |
| SPATA5 |  |  | 7 | 7 |
| SPATA5L1 |  |  | 9 | 10 |
| STARD13 |  |  | 3 | 4 |
| STAT3 | 5 | 7 | 5 | 5 |
| STIP1 | 4 | 4 |  |  |
| STK3 | 3 | 3 | 3 | 3 |
| STRN3 | 10 | 10 |  |  |
| STRN4 | 5 | 5 |  |  |
| SUPT5H | 11 | 12 |  |  |
| SYBU | 3 | 3 |  |  |
| TADA3 | 9 | 13 | 3 | 4 |
| TANC2 | 22 | 23 |  |  |
| TBC1D4 | 3 | 4 | 4 | 5 |
| TBL3 |  |  | 3 | 4 |
| TCF25 | 3 | 3 | 3 | 3 |
| TEAD1 | 10 | 11 | 9 | 13 |
| TEAD3 | 5 | 8 | 4 | 4 |
| TET2 | 4 | 5 | 5 | 5 |
| TNIP1 | 3 | 4 |  |  |
| TPR | 4 | 4 |  |  |
| TRADD | 3 | 3 |  |  |
| TRAP1 | 12 | 14 | 12 | 15 |
| TRIM37 | 12 | 13 | 9 | 9 |
| TRIP13 |  |  | 4 | 4 |
| TRIP6 | 6 | 10 | 5 | 6 |
| TRMT1 | 4 | 5 |  |  |
| TRMT61B | 6 | 6 |  |  |
| TSEN2 | 6 | 6 |  |  |
| TSEN54 | 12 | 14 | 9 | 10 |
| TSG101 | 3 | 3 |  |  |
| TSR1 |  |  | 8 | 9 |
| TTK | 3 | 3 |  |  |
| U2SURP |  |  | 3 | 3 |
| UBE3A | 5 | 5 | 3 | 3 |
| UBE3C |  |  | 6 | 6 |
| UBR2 | 9 | 11 |  |  |
| UBR5 | 13 | 14 | 28 | 29 |
| UNK | 3 | 3 |  |  |
| VBP1 | 14 | 27 | 12 | 33 |
| VCPIP1 | 10 | 12 | 5 | 5 |
| VPS11 | 3 | 3 |  |  |
| YAP1 | 15 | 17 | 9 | 9 |
| YLPM1 | 8 | 10 | 4 | 5 |
| ZC3H14 | 3 | 3 | 5 | 5 |
| ZNHIT2 | 8 | 13 | 8 | 14 |
